# OTU deubiquitinase 5 inhibits the progression of non‐small cell lung cancer via regulating p53 and PDCD5

**DOI:** 10.1111/cbdd.13688

**Published:** 2020-04-19

**Authors:** Xiao‐yun Kang, Jing Zhang, Ling Tang, Liu Huang, Jin Tong, Qiang Fu

**Affiliations:** ^1^ Department of Oncology Tongji Hospital Tongji Medical College Huazhong University of Science and Technology Wuhan China; ^2^ Department of Oncology Xinfeng County People's Hospital Xinfeng China; ^3^ Department of PICC Tongji Hospital Tongji Medical College Huazhong University of Science and Technology Wuhan China

**Keywords:** NSCLC, OTUD5, p53, PDCD5

## Abstract

Non‐small cell lung cancer (NSCLC) has the highest morbidity and mortality worldwide. OTU deubiquitinase 5 (OTUD5), a deubiquitinating enzyme, can enhance the stability of p53 and programmed cell death 5 (PDCD5), a protein related to the apoptosis, by deubiquitination. This study aimed to explore the biological function and underlying mechanism of OTUD5 in NSCLC. Western blot and qRT‐PCR were used to detect the expression of OTUD5 protein and mRNA in NSCLC tissues and cells, respectively. RNAi was adopted to construct an OTUD5 low‐expression model while the plasmids overexpressing p53 and PDCD5 were used to establish the overexpression models, respectively. CCK‐8 assay, transwell assay, and apoptosis assay were carried out to analyze the changes in the proliferation, migration, and chemoresistance of A549 and HCC827 cells. The mechanism of OTUD5 in NSCLC was studied by Western blot. Down‐regulated OTUD5 in NSCLC tissues was significantly correlated to a poor prognosis. The knockdown of OTUD5 inactivated p53 and PDCD5, promoting the proliferation and metastasis of NSCLC cells while inhibiting their apoptosis. OTUD5 knockdown also enhanced the resistance of NSCLC cells to doxorubicin and cisplatin. OTUD5 acted as a tumor suppressor in NSCLC by regulating the p53 and PDCD5 pathways.

## Graphical abstract

OTUD5 is down‐regulated in NSCLC tissues, and it inhibits the malignant phenotypes of NSCLC cells via regulating p53 and PDCD5. Its knockdown increases the sensitivity of NSCLC cells to cisplatin and doxorubicin, and it may be a promising target for NSCLC therapy.

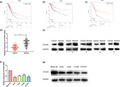

## 1 INTRODUCTION

As the most common malignant tumor, lung cancer has the highest morbidity and mortality in the world, and 1.2 million people die of lung cancer every year (Molina, Yang, Cassivi, Schild, & Adjei, 2008). Among various types of lung cancer, non‐small cell lung cancer (NSCLC) accounts for 80%~85% of the total number of cases, and most NSCLC patients have missed the best chance for surgery at the time of diagnosis. In addition, more than 40% of NSCLC patients treated by surgery will experience recurrence (Molina et al., 2008). Therefore, by exploring the pathogenesis of NSCLC, it may help the development of new targeted drugs to improve the survival of NSCLC patients experiencing metastasis or recurrence.

As a kind of deubiquitinating enzyme, OTU deubiquitinase 5 (OTUD5) has been proven to regulate innate immune responses, and its primary mechanism is to regulate the ubiquitination of type I interferon adaptor proteins (Huang et al., 2012; Kayagaki et al., 2007). It was reported that OTUD5 can deubiquitinate p53, an important stress sensor, to improve its stability (Luo et al., 2013). When cells are subject to internal and external stimuli, the stability of p53 rapidly changes. P53 exerts its anti‐cancer effects by inducing the expression of downstream genes, maintaining the genomic stability, and inducing apoptosis. P53 mutations or deletions can lead to the loss of p53 functions, promoting the malignant transformation of cells (Chen, Chen, Bookstein, & Lee, 1990). Considering the tumor‐suppressive function of p53, it was hypothesized here that OTUD5 also acted as a tumor suppressor.

P53 mutations occur in about 80% of tissue samples from lung cancer patients (Cancer Genome Atlas Research Network, 2012), suggesting that the role of OTUD5 as a tumor suppressor may not solely depend on the regulation of p53. Studies have shown that OTUD5 can activate the programmed cell death 5 (PDCD5, a tumor suppressor; Park et al., 2015). In addition to regulating the stability of p53, PDCD5 also inhibits the progression of tumors through other mechanisms (Park et al., 2015). On one hand, PDCD5 regulates the transfer of Bax protein to the mitochondrial outer membrane, resulting in a decrease in membrane potential and the release of cytochrome C, thereby initiating the apoptotic process (Essers et al., 2015); on the other hand, PDCD5 binds to caspase‐3 and prolongs the half‐life of activated caspase‐3 to exert a positive regulatory effect (Li et al., 2012). PDCD5 shows a higher mutation rate in lung cancer patients with shorter survival (Spinola et al., 2006). It was also reported that as compared with that in normal lung tissues, PDCD5 expression was significantly decreased in highly differentiated lung adenocarcinoma tissues (Xu, Sui, Yuan, & Zou, 2014). These studies indicate that PDCD5 plays an important role in the progression of NSCLC. Therefore, it is worth exploring whether OTUD5 can affect the progression of NSCLC, especially in cases carrying p53 mutations, by regulating PDCD5.

In this study, it was demonstrated that OTUD5 was down‐regulated in NSCLC tissues. In vitro experiments validated the tumor‐suppressive function of OTUD5 in NSCLC. Additionally, OTUD5 was found to regulate the expression of p53 and PDCD5. The chemosensitivity test proved that OTUD5 affected the resistance of NSCLC cells to cisplatin and doxorubicin. This study firstly validated the anti‐tumor function of OTUD5 in NSCLC and provided new clues for the development of new NSCLC therapies.

## 2 MATERIALS AND METHODS

### 2.1 Tissue collection

A total of 95 samples of NSCLC tissues and adjacent normal tissues were randomly collected from patients visiting the Tongji Hospital (Wuhan, China) from 2014 to 2018. All patients gave informed consent to the study participation. The specimens were collected and used under the approval of the Ethics Review Committee of the Tongji Hospital. No patients in this study received neoadjuvant therapy (chemotherapy or radiotherapy) before surgical operations. The resected tissues were immediately frozen after resection and stored in liquid nitrogen at −196°C for subsequent tests.

### 2.2 Cell culture

Human NSCLC cell lines A549 (wild type p53), H358 (p53 null), H1299 (p53 null), and HCC827 (mutant type p53), and human bronchial epithelial cell line BEAS‐2B were purchased from American Type Culture Collection (ATCC). All NSCLC cell lines were cultured in a Dulbecco modified Eagle's medium or an RPMI‐1640 medium (YajiBio, Minhang) supplemented with 10% fetal bovine serum (FBS, Invitrogen), 100 U/ml of penicillin, and 100 μg/ml of streptomycin (Invitrogen). The cells were cultured in 5% CO_2_ and 90% humidified air at 37°C. BEAS‐2B was cultured in a Bronchial Epithelial Basal Medium (BEBM, LONZA) supplemented with 0.1% recombinant human epidermal growth factor and 0.1% insulin.

### 2.3 Cell transfection

OTUD5 siRNA, negative control siRNA, p53 overexpressing plasmid, and PDCD5 overexpressing plasmid were constructed by RiboBio Co. Ltd. NSCLC cells were planted into six‐well plates at the density of 1.5 × 10^5^ per/well, and the siRNAs or plasmids were transfected into the cells using Lipofectamine^®^ 3000 (Invitrogen). After 48 hr, the cells were harvested for further testing.

### 2.4 Quantitative real‐time polymerase chain reaction (qRT‐PCR)

Total RNA was extracted by using a TRIzol reagent (Invitrogen). A synthesis kit (TOYOBO) was used to synthesize the first strand cDNA from l μg of total RNA. Afterward, an ABI 7500 Real‐Time PCR System (Applied Biosystems) was adopted to carry out the amplification and quantitative analysis of the prepared cDNA samples using a Power SYBR Green PCR Master Mix (Applied Biosystems). The primer sequences were shown as follows:

OTUD5: (forward) 5′‐TCCACAAGAGCCAAGGCAT‐3′;
(reverse) 5’‐GTGGCATAGAAGTGCAGCA‐3′.
P53: (forward) 5′‐ACTGCATGGACGATCTGTTG‐3′;
(reverse) 5′‐GTGACAGGGTCCTGTGCTG‐3′.
PDCD5: (forward) 5′‐ACAGATGGCAAGATATGGACA‐3′;
(reverse) 5′‐TCCTAGACTTGTTCCGTTAAG‐3′.
GAPDH: (forward) 5′‐CAGGAGGCATTGCTGATGAT‐3′;
(reverse) 5′‐GAAGGCTGGGGCTCATTT‐3′.

### 2.5 Western blot

The cells were washed for three times with PBS. Then, the total protein was extracted using RIPA lysate (Beyotime Biotechnology) containing protease inhibitors (Roche). After the electrophoretic separation was carried out using a 10% SDS‐PAGE gel, the proteins were transferred to a PVDF membrane. After being blocked by 5% skim milk, the PVDF membrane was incubated with primary antibodies at 4°C overnight. The primary antibodies included anti‐OTUD5 (D8Y2U, Cat. No. 20087; Cell Signaling Technology), anti‐PDCD5 (1:1,000, Rabbit, ab126213, Abcam), and anti‐p53 (1:1,000, Rabbit, ab131442, Abcam) antibodies. On the next day, the PVDF membrane was washed with PBS and incubated with horseradish peroxidase‐labeled secondary anti‐rabbit antibodies at room temperature for 1.5 hr. Ultimately, an enhanced chemiluminescent Western blot detection kit (Engreen Biossystem) was used to detect protein bands.

### 2.6 Transwell assay

In accordance with the manufacturer's instructions, a 24‐well plate with Transwell chambers (8 μm pore diameter; BD Biosciences) was used to measure the migration and invasion capacity of cells. The transfected cells (5 × 10^4^ cells/ml) were added with a serum‐free medium to the upper chamber. Then, 450 µl of medium containing 10% FBS was added to the lower chamber. Twenty‐four hours later, the cells on the upper surface of the transwell chambers were gently wiped off with a cotton swab. The migrated or invaded cells were fixed with 4% paraformaldehyde and then stained with crystal violet before they were counted under a microscope.

### 2.7 CCK‐8 assay and IC_50_ measurement

The cell viability was determined by the CCK‐8 method. In short, the untreated cells (5 × 10^3^) were inoculated into each well of a 96‐well plate. At 24 hr after the inoculation, different concentrations of cisplatin (DDP) or doxorubicin were added and the cells were further incubated for 48 hr. Afterward, 10 μl of CCK‐8 solution (Dojindo Laboratories) was added to each well to continue the incubation in 5% CO_2_ and at 37°C for 1 hr. In the end, a microplate reader (BioTek) was used to measure the absorbance at a 450 nm wavelength. IC_50_ was measured as the concentration of the drugs producing 50% of growth inhibition. A higher IC_50_ value indicated a stronger drug resistance. The experiment was repeated three times.

### 2.8 Flow cytometry

The untreated cells were trypsinized, resuspended, and stained with an AnnexinV‐PE kit (Beyotime) along with a propidium iodide solution (Beyotime). Ultimately, a flow cytometry analysis was performed using Cytoflex (Beckman Coulter).

### 2.9 Statistical analysis

SPSS20.0 (SPSS, Inc.) and Graphpad Prism7 were adopted for statistical analysis. All data were expressed as mean value ± standard deviations. The *t* test was carried out to analyze the differences between two groups. The chi‐square test was performed to calculate the relationship between OTUD5 and clinicopathological features. A *p‐*value of <.05 indicated statistical differences.

## 3 RESULTS

### 3.1 Reduced OTUD5 expression was related to a poor prognosis

**Figure 1:**
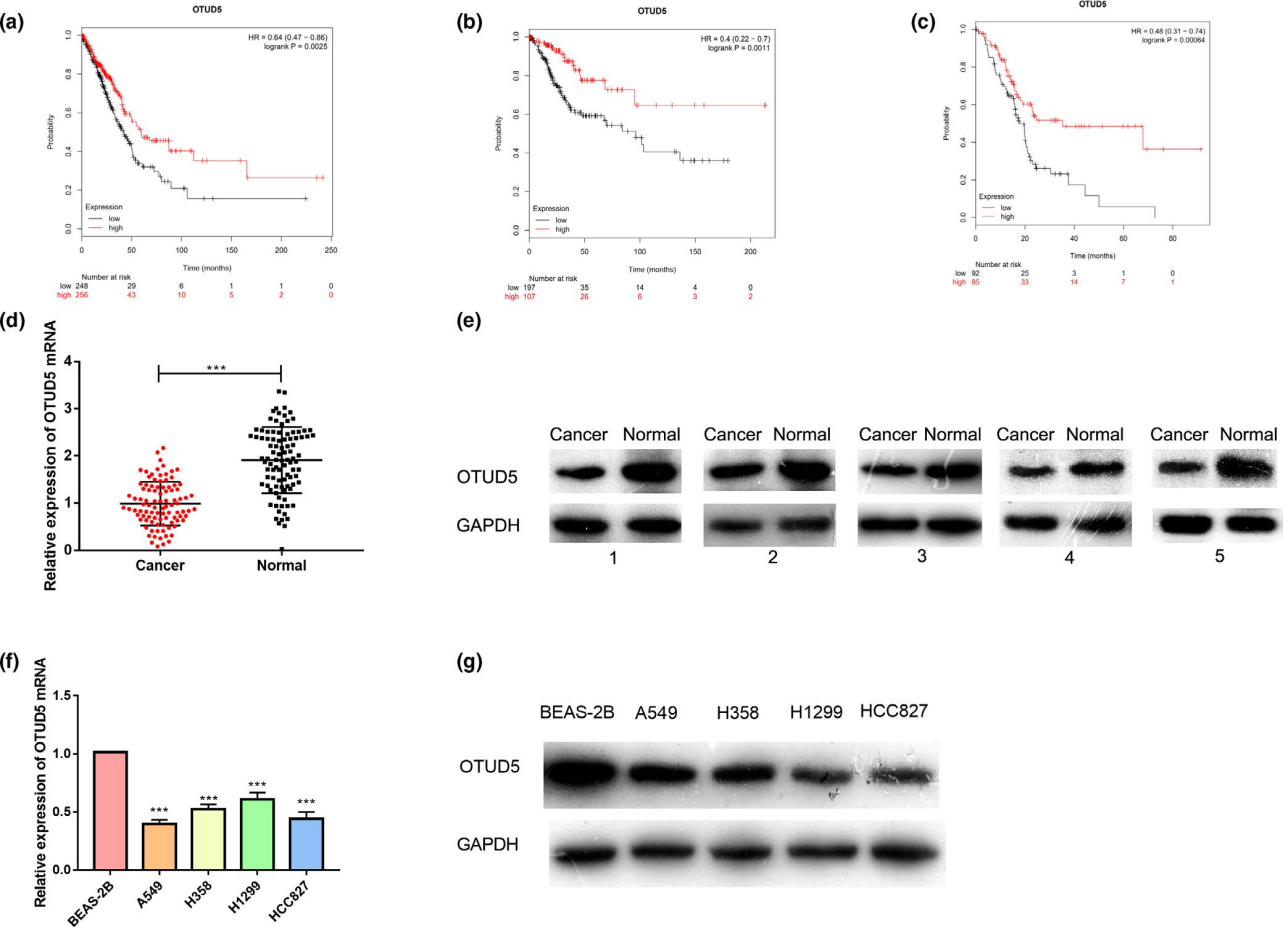
The expression of OTUD5 was reduced in NSCLC. The Kaplan–Meier method was used to analyze the survival time of patients with lung adenocarcinoma (a), pancreatic cancer (b), and cervical squamous carcinoma (c) who showed high or low expression of OTUD5. (d–e) RT‐PCR and Western blot were applied to detect the expression of OTUD5 mRNA and protein in tumor tissues and adjacent normal tissues, respectively. ***p < .001. (f–g) RT‐PCR and Western blot were applied to detect the expression of OTUD5 mRNA and protein in NSCLC cell lines A549, H358, H1299, and HCC827, and human bronchial epithelial cell line BEAS‐2B. ***p < .001 [Colour figure can be viewed at wileyonlinelibrary.com]

In order to understand the relationship between OTUD5 expression and cancers, the survival curves of patients with lung adenocarcinoma, pancreatic cancer, and cervical squamous carcinoma were analyzed first by using the KM plotter (http://kmplot.com/analysis/). The results suggested that, in these three cancers, the patients with low OTUD5 expression had a shorter survival (*p* < .05, log‐rank test, Figure 1a–c). These results suggested that OTUD5 could act as an indicator for favorable prognosis in cancer. To determine the specific clinical significance of OTUD5 in NSCLC, the relationship between OTUD5 expression and the clinical features of NSCLC was also assessed. According to the OTUD5 expression in NSCLC tissues detected by qRT‐PCR, the 95 patients were divided into a low‐expression group (*n* = 47) and a high‐expression group (*n* = 48). The results showed that lower OTUD5 expression was related to worse tumor differentiation, larger tumor size, and lymphatic metastasis, but the OTUD5 expression showed no association with age, gender, smoking history, and TNM staging of NSCLC (Table 1).

**Table 1:** Correlation between OTUD5 expression and pathological parameters in NSCLC

Pathological parameters	No. of patients	OTUD5 expression		Chi‐square	*p‐*value
		Low	High		
All patients	95	47	48		
Age				0.0117	.9139
<60	50	25	25		
≥60	45	22	23		
Gender				1.5743	.2096
Male	65	35	30		
Female	30	12	18		
Smoking status				1.8202	.1773
Smokers	59	26	33		
Non‐smokers	36	21	15		
Tumor size (cm)				4.8186	.0282
≥3	36	23	13		
<3	59	24	35		
TNM stage				0.5053	.4772
I + II	54	25	29		
III + IV	41	22	19		
Differentiation				10.4815	.0012
Well and moderate	40	12	28		
Poor	55	35	20		
Lymphatic metastasis				4.7270	.0297
No	43	16	27		
Yes	52	31	21		

Then, qRT‐PCR was carried out to compare the expression of OTUD5 mRNA in NSCLC tissues and adjacent normal tissues collected from the 95 patients. The results showed that the expression of OTUD5 in NSCLC tissues was significantly lower than that in corresponding non‐tumor tissues (*p* < .001, Figure 1d). Western blot was also used to detect the OTUD5 protein expression in five pairs of NSCLC tissues/normal tissues, and the results validated that the protein expression of OTUD5 was also reduced in NSCLC tissues (Figure 1e). In order to verify the above results, the expression of OTUD5 was studied in NSCLC cell lines A549, H358, H1299, and HCC827 and human bronchial epithelial cell line BEAS‐2B. As shown in the results, the OTUD5 expression in NSCLC cell lines was significantly down‐regulated compared with that of BEAS‐2B (*p* < .001, Figure 1f,g). Collectively, these data indicated that OTUD5 was down‐regulated in NSCLC tissues and cells.

### 3.2 Knockdown of OTUD5 accelerated the proliferation, migration, and invasion of A549 cells, and inhibited their apoptosis

**Figure 2:**
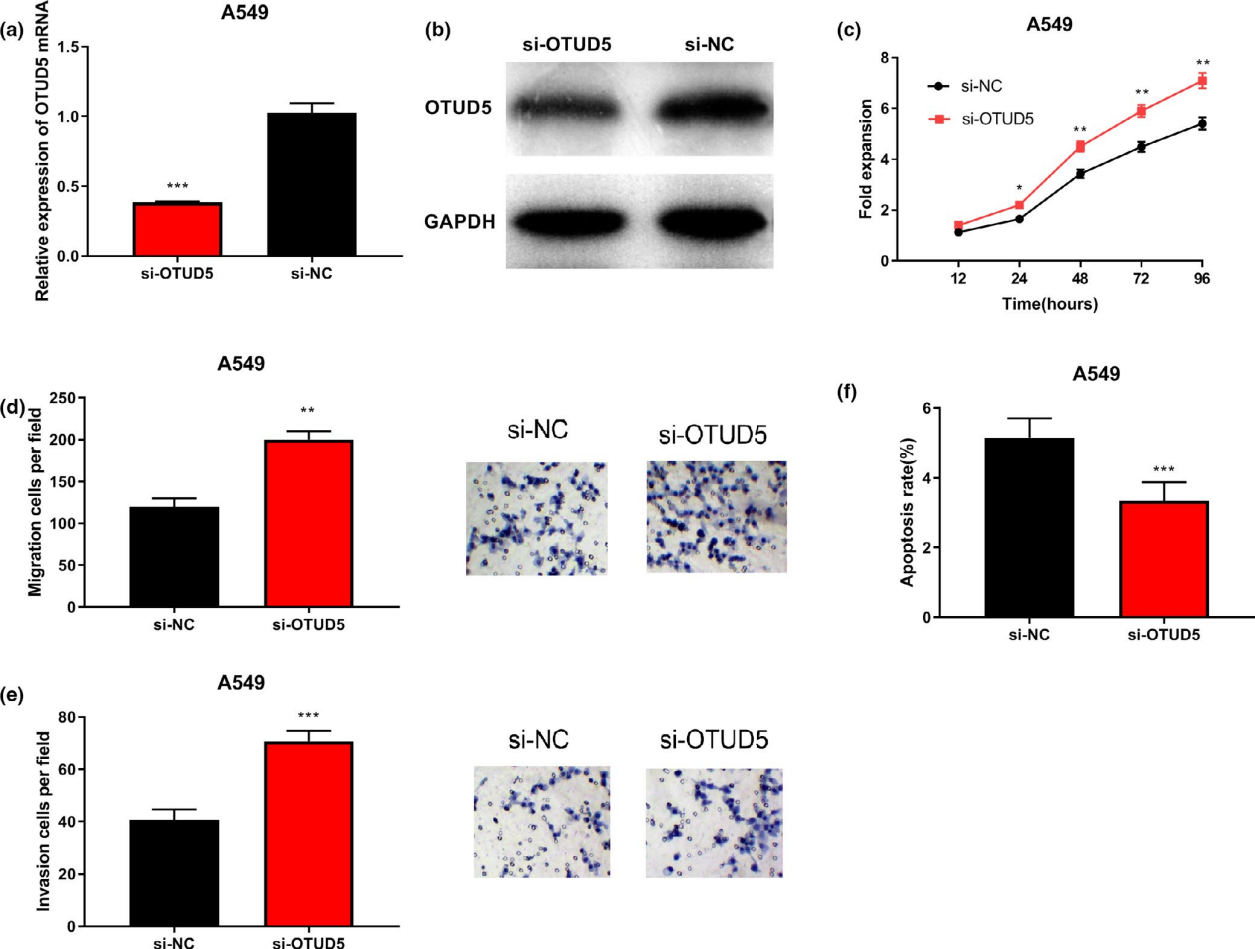
Knockdown of OTUD5 accelerated the proliferation, migration, and invasion of A549 cells while inhibiting their apoptosis. (a–b) RT‐PCR and Western blot were applied to detect OTUD5 mRNA and protein expression in A549 cells transfected with OTUD5 siRNA and NC siRNA. ***p < .001. CCK‐8 assay, transwell assay, and apoptosis analysis were carried out to detect the proliferation (c), migration (d), invasion (e), and apoptosis (f) of A549 cells, respectively. *p < .05, **p < .01, and ***p < .001 [Colour figure can be viewed at wileyonlinelibrary.com]

Next, A549 cells were used for the following experiment. In order to explore the biological effects of OTUD5 on NSCLC cells, OTUD5 siRNA and NC siRNA were transfected into A549 cells, and a model with low OTUD5 expression was successfully constructed (*p* < .001, Figure 2a,b). Cell proliferation was detected by the CCK‐8 method, and the results indicated that the proliferative ability of A549 cells in the si‐OTUD5 group was significantly enhanced compared with that in the si‐NC group (*p* < .05, Figure 2c). Transwell assays were performed to detect cell migration and cell invasion, and the results indicated that the number of both migrating and invading cells in the si‐OTUD5 group was significantly increased compared with that in the si‐NC group (*p* < .05, Figure 2d,e). Cell apoptosis was detected by flow cytometry, and the results showed that the apoptosis rate of the cells in the si‐NC group was significantly lower than that of the OTUD5 knockdown group (*p* < .001, Figure 2f). Hence, it was concluded that the down‐regulation of OTUD5 strengthened the proliferation, invasion, and migration of A549 cells while inhibiting their apoptosis.

### 3.3 OTUD5 impeded the progression of NSCLC cells via regulating p53

**Figure 3:**
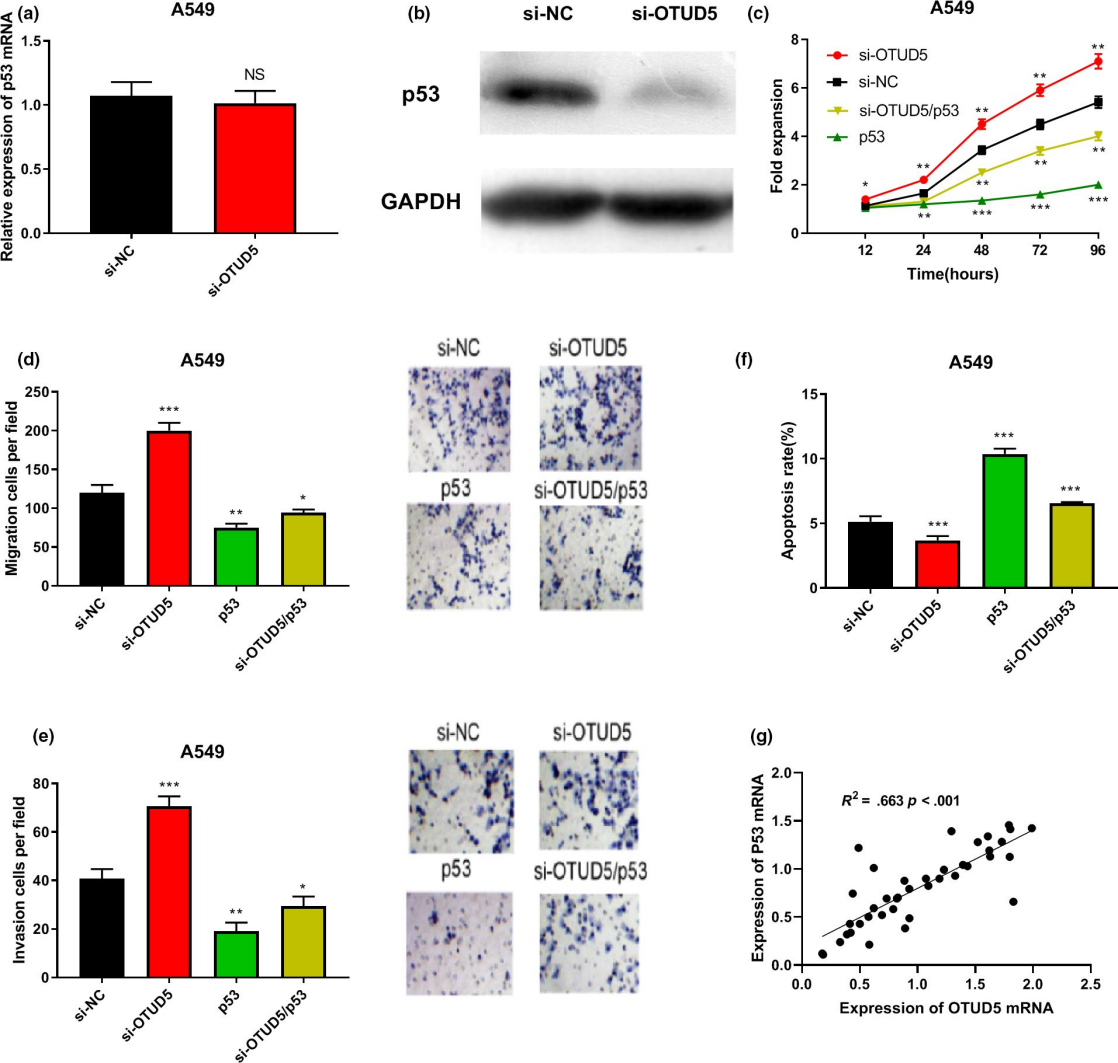
OTUD5 impeded the progression of NSCLC cells via regulating p53. (a) Western blot was applied to measure p53 expression in A549 cells transfected with OTUD5 siRNA and NC siRNA. (b) RT‐PCR was applied to measure p53 mRNA expression in A549 cells transfected with OTUD5 siRNA and NC siRNA. CCK‐8 assay, transwell assay, and apoptosis analysis were carried out to detect the proliferation (c), migration (d), invasion (e), and apoptosis (f) of A549 cells, respectively. *p < .05, **p < .01, and ***p < .001. (g) The Pearson correlation test was used to analyze the correlation between OTUD5 and p53 expression in NSCLC samples [Colour figure can be viewed at wileyonlinelibrary.com]

In order to study whether OTUD5 regulates p53 expression in NSCLC, OTUD5 siRNA and NC siRNA were transfected into A549 cells, respectively. It was indicated that the expression of p53 protein was decreased significantly while the expression of p53 mRNA was not obviously changed in the cells with knocked down OTUD5 expression (*p* > .05, Figure 3a,b). This result implied that p53 expression was modulated by post‐translational regulation of OTUD5, which was consistent with the role of OTUD5 as a deubiquitinating enzyme. In order to study whether OTUD5 can inhibit NSCLC via regulating p53 expression, OTUD5 siRNA and a p53 overexpressing plasmid were used to construct an si‐OTUD5/p53 group, an si‐OTUD5 group, a p53 group, and an si‐NC group, respectively. The CCK‐8 assay showed that the overexpression of p53 partly offsets the effect of OTUD5 knockdown on promoting cell proliferation (*p* < .05, Figure 3c). The transwell assay suggested that the number of migrating and invading cells in the si‐OTUD5/p53 group was significantly lower than that in the si‐OTUD5 group (*p* < .05, Figure 3d,e). The apoptosis assay showed that the overexpression of p53 partly counteracted the inhibitory effect of si‐OTUD5 on apoptosis (*p* < .05, Figure 3f). Then, tissue samples were randomly collected from 40 NSCLC patients and the Pearson correlation test was used to detect the correlation between the expression of OTUD5 mRNA and p53 mRNA. The results showed that in NSCLC tissues, there was a positive correlation between the expression of OTUD5 mRNA and p53 mRNA (*p* < .001, Figure 3g, *R* ^2^ = 0.663). Therefore, it was concluded that OTUD5 impeded the transformation of malignant phenotypes of A549 cells via positively regulating p53 expression.

### 3.4 OTUD5 regulated the proliferation, metastasis, and apoptosis of p53‐mutant NSCLC cells via modulating PDCD5 expression

**Figure 4:**
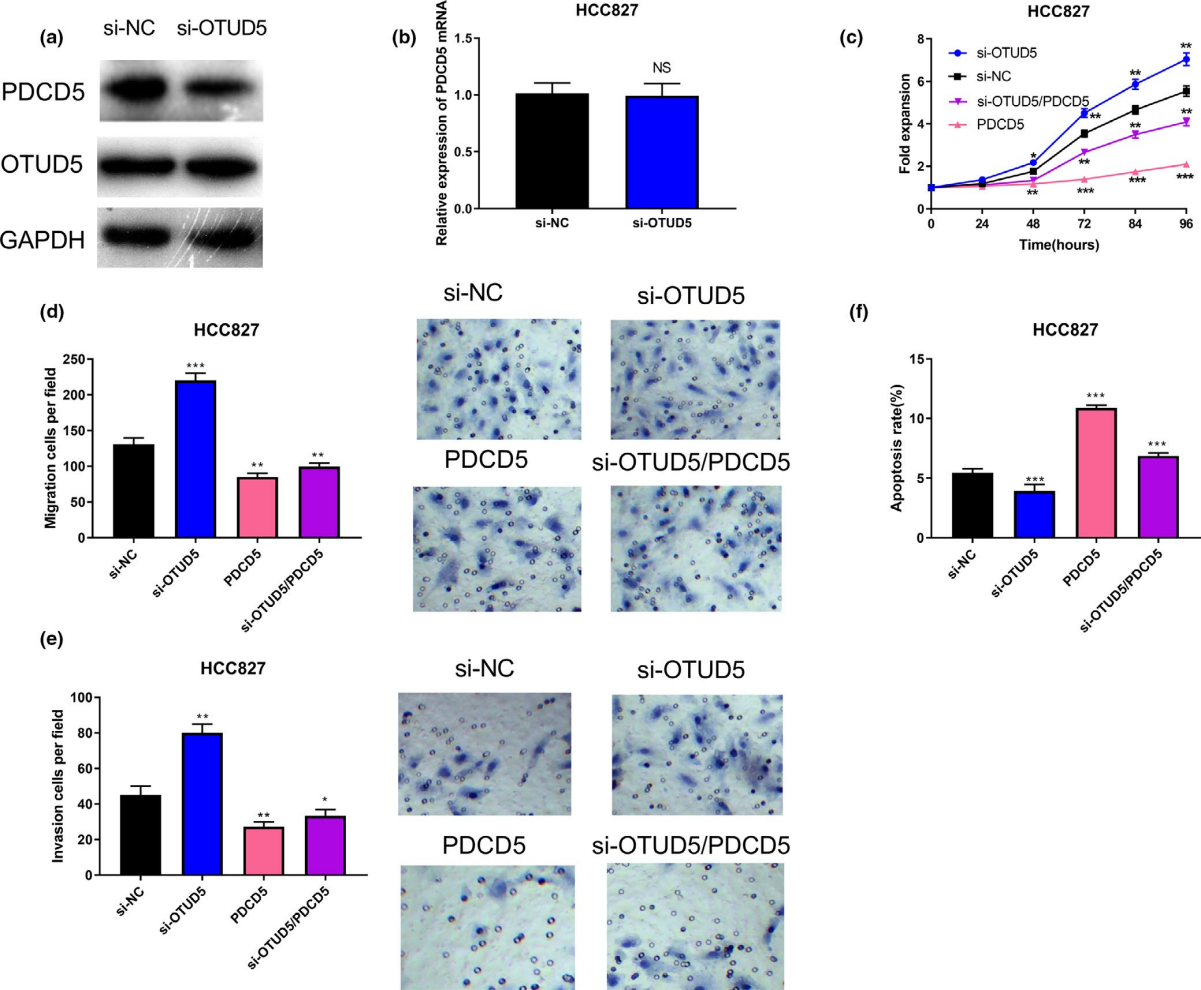
Knockdown of OTUD5 promoted the proliferation, migration, and invasion of HCC827 cells while inhibiting their apoptosis via regulating PDCD5. Western blot (a) and RT‐PCR (b) were used to detect PDCD5 expression in HCC827 cells transfected with OTUD5 siRNA and NC siRNA. CCK‐8 assay, transwell assay, and apoptosis analysis were carried out to detect the proliferation (c), migration (d), invasion (e), and apoptosis (f) of HCC827 cells, respectively. *p < .05, **p < .01, and ***p < .001 [Colour figure can be viewed at wileyonlinelibrary.com]

In order to study whether OTUD5 can regulate PDCD5 expression in p53 mutant NSCLC cells, OTUD5 siRNA and NC siRNA were transfected into HCC827 cells, respectively. Western blot and qRT‐PCR were used to detect the expression of PDCD5, and it was indicated that the protein expression of PDCD5 was significantly reduced in cells with down‐regulated OTUD5 expression, but the mRNA expression of PDCD5 displayed no obvious changes (*p* > .05, Figure 4a,b). To explore whether OTUD5 could exert its anti‐cancer effects on NSCLC via regulating PDCD5 expression, OTUD5 siRNA and the PDCD5 overexpressing plasmid were used to construct an si‐OTUD5/PDCD5 group, an si‐OTUD5 group, a PDCD5 group, and an si‐NC group, respectively. The CCK‐8 assay showed that the overexpression of PDCD5 partly offsets the effects of OTUD5 knockdown on promoting cell proliferation (*p* < .05, Figure 4c). The transwell assay indicated that the number of migrating and invading cells in the si‐OTUD5/PDCD5 group was significantly decreased compared with that in the si‐OTUD5 group (*p* < .05, Figure 4d,e). Moreover, the apoptosis assay showed that the overexpression of PDCD5 partly offsets the inhibitory effect of si‐OTUD5 on cell apoptosis (*p* < .001, Figure 4f). Therefore, it was concluded that OTUD5 could impede the proliferation, migration, and invasion of HCC827 cells while facilitating their apoptosis, and the role of OTUD5 was partly mediated by PDCD5.

### 3.5 OTUD5 regulated the resistance of NSCLC cells to cisplatin and doxorubicin

**Figure 5:**
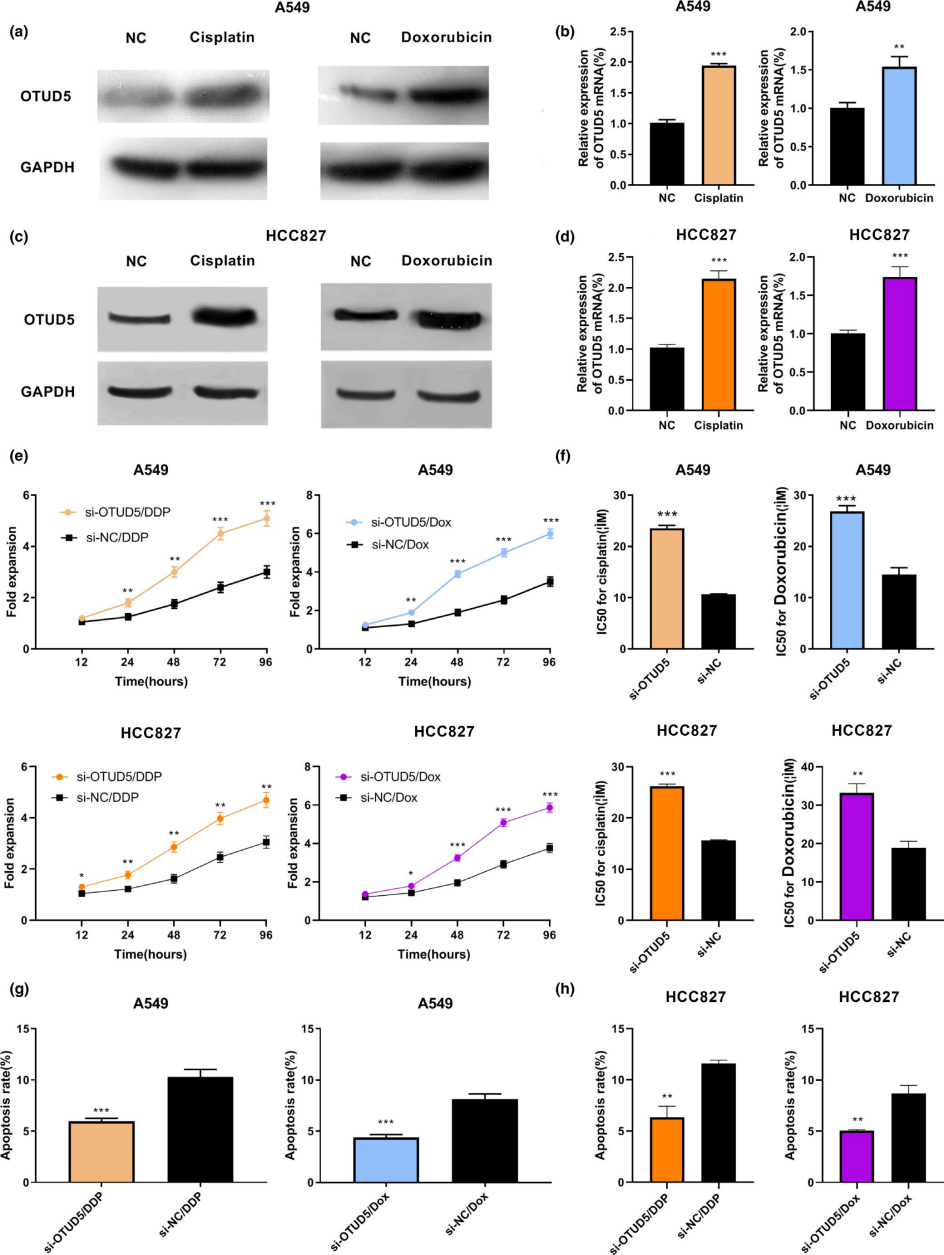
OTUD5 affected the resistance of NSCLC cells to cisplatin and doxorubicin. (a–d) The expression of OTUD5 in A549 and HCC827 cells treated with cisplatin and doxorubicin was detected by Western blot and RT‐PCR. **p < .01 and ***p < .001. (e) After cisplatin and doxorubicin treatments, the CCK‐8 assay was performed to detect the viability of A549 and HCC827 cells transfected with OTUD5 siRNA or NC siRNA. *p < .05, **p < .01, and ***p < .001. (f) The IC_50_ values of cisplatin and doxorubicin in NSCLC cells were compared before and after A549 and HCC827 cells were transfected with OTUD5 siRNA or NC siRNA. **p < .01 and ***p < .001. After cisplatin and doxorubicin treatments, flow cytometry analysis was used to detect the apoptosis of A549 (g) and HCC827 (h) cells transfected with OTUD5 siRNA or NC siRNA. **p < .01 and ***p < .001 [Colour figure can be viewed at wileyonlinelibrary.com]

Since OTUD5 played a vital role in cell proliferation, migration, and apoptosis, the next step was to explore whether OTUD5 was related to the chemosensitivity of NSCLC cells. It was found that both cisplatin and doxorubicin significantly increased the expression of OTUD5 in A549 and HCC827 cells (*p* < .01, Figure 5a,d). In order to assess the function of OTUD5 in regulating the resistance to cisplatin and doxorubicin, the CCK‐8 assay was used to detect the viability of A549 and HCC827 cells treated with different concentration of cisplatin and doxorubicin. It was found that the viability of cells in the OTUD5 knockdown group was higher than that in the NC group (*p* < .05, Figure 5e,f). After the treatment with cisplatin or doxorubicin, the apoptosis of A549 and HCC827 cells in the OTUD5 knockdown group was reduced compared with that in the control group (*p* < .01, Figure 5g,h). All these results suggested that OTUD5 played a crucial role in the resistance of NSCLC cells to cisplatin and doxorubicin.

## 4 DISCUSSION

In the present study, the expression of OTUD5 in NSCLC tissues was detected, and the results showed that OTUD5 expression in NSCLC tissues was significantly down‐regulated and was correlated with adverse pathological parameters of the patients. It was also demonstrated that OTUD5 could affect the proliferation and metastasis of NSCLC cells. For the first time, it was also suggested in this study that OTUD5 acted as a tumor suppressor in NSCLC. The results also suggested that low OTUD5 expression was associated with the poor prognosis in other types of tumors (pancreatic cancer, cervical cancer, etc.), but the function of OTUD5 in other types of tumors remains to be clarified.

Ubiquitination is an important process for intracellular protein degradation. With the help of the ubiquitin‐activating enzyme E1 and ubiquitin‐conjugating enzyme E2, ubiquitin molecules are attached to the substrates via ubiquitinated ligase E3. The ubiquitin‐linked proteins are recognized and degraded by unique ubiquitination proteasomes in cells, and this type of degradation is generally carried out in the cytosol (Joazeiro & Weissman, 2000; Livneh, Cohen‐Kaplan, Cohen‐Rosenzweig, Avni, & Ciechanover, 2016; Pickart & Eddins, 2004; Roos‐Mattjus & Sistonen, 2004). The presence of this intracellular protein degradation system enables the cells to effectively regulate the level of related proteins. Accumulating evidence has indicated that ubiquitin ligases and deubiquitinases are involved in regulating cancer progression. For example, deubiquitinase otubain 1 regulates many cancer‐associated signaling pathways, including MAPK, mTORC1, and FOXM1, to promote cancer progression (Saldana, VanderVorst, Berg, Lee, & Carraway, 2019); the E3 ubiquitin ligase Hakai promoted the proliferation, metastasis, and chemoresistance of NSCLC cells (Liu, Wu, Tao, & Ma, 2018). P53 is an important protein involved in cell cycle regulation, while Mdm2, E6/E6AP, P300, Pirh2, COP1, and other proteins are involved in the regulation of ubiquitination and degradation of p53 (Jung, Qian, & Chen, 2012; Kwon, Saindane, & Baek, 2017). It was shown that ubiquitinated p53 proteins were eventually degraded in the cytoplasm by the protein ubiquitination system, thereby achieving the negative regulation of p53 proteins (Livneh et al., 2016; Roos‐Mattjus & Sistonen, 2004). Previous studies have shown that OTUD5 can regulate the cellular expression of p53 by regulating its deubiquitination (Luo et al., 2013). This study demonstrated that OTUD5 could inhibit the transformation of malignant phenotypes of A549 cells by inducing the expression of p53, suggesting that OTUD5 may be used as a potential therapeutic target for NSCLC treatment.

PDCD5 is a member of the family of programmed cell death proteins, which are often involved in tumor growth and cell apoptosis (Li et al., 2018). For example, PDCD5 inhibits the metastasis of osteogenicsarcoma cells via regulating the TGF‐β1/Smad signaling pathway, which is related to a favorable prognosis (Zhao et al., 2019). The overexpression of PDCD5 sensitizes hepatocellular carcinoma cells to cisplatins and reduces cell invasion (Fan, Yao, Yao, & Li, 2015)]. In glioma, the expression of PDCD5 was significantly reduced in highly malignant cases, making PDCD5 a tumor suppressor (Wang, Li, Li, & Gong, 2018). In this study, it was demonstrated that OTUD5 impeded the proliferation and metastasis of the NSCLC cell line HCC827 via regulating PDCD5 expression. These results indicated the vital tumor‐suppressive function of the OTUD5/PDCD5 axis in the progression of NSCLC, especially in patients carrying mutated p53.

Chemoradiotherapy plays a vital role in the treatment of lung cancer. Cisplatin‐based combination chemotherapy (e.g., combined with doxorubicin) significantly improves the clinical outcomes in NSCLC treatment, but drug resistance commonly contributes to disease progression and relapse. In this study, it was found that the inhibition of OTUD5 could promote the resistance of NSCLC cells to chemotherapeutic drugs such as doxorubicin and cisplatin. A recent study demonstrated that OTUD5 could increase the radiosensitivity of cervical cancer cells (Yin et al., 2019), which was consistent with the results of this study. Studies have shown that OTUD5 could inhibit DNA damage repair by regulating the FACT histone chaperone complex (de Vivo et al., 2019). Since the FACT histone chaperone complex can promote the resistance of tumor cells to DNA damage factors (Dejmek, Iglehart, & Lazaro, 2009; Koman et al., 2012; Yarnell, Oh, Reinberg, & Lippard, 2001), a follow‐up research is necessary to determine whether OTUD5 can reduce chemoradiotherapy resistance by antagonizing the FACT histone chaperone complex.

In summary, it was found in this study that the down‐regulation of OTUD5 was related to a poor prognosis in NSCLC patients. It was proved that OTUD5 inhibited the proliferation and metastasis of NSCLC cells but promoted their apoptosis via regulating p53 and PDCD5. In addition, OTUD5 could regulate the sensitivity of NSCLC cells to cisplatin and doxorubicin. All these data revealed the importance of OTUD5 in the development of NSCLC, and OTUD5 may become a new therapeutic target for NSCLC treatment.

## CONFLICT OF INTEREST

The authors declare that they have no competing interest.

## AUTHORS’ CONTRIBUTION

XYK and JZ conceived and designed the experiments. XYK, JZ, and LT performed the experiments. XYK and JZ contributed to statistical analysis. XYK, JZ, TL, LH, JT, and QF wrote the paper. All authors read and approved the final manuscript.

## Data Availability

The data used to support the findings of this study are available from the corresponding author upon request.
